# Immunogenicity Differences of the ChAdOx1 nCoV-19 Vaccine According to Pre-Existing Adenovirus Immunity

**DOI:** 10.3390/vaccines11040784

**Published:** 2023-04-01

**Authors:** Jinnam Kim, Changhyup Kim, Jung Ah Lee, Se Ju Lee, Ki Hyun Lee, Jung Ho Kim, Jin Young Ahn, Su Jin Jeong, Nam Su Ku, Joon-Sup Yeom, Young Goo Song, Jun Yong Choi

**Affiliations:** 1 Division of Infectious Diseases, Department of Internal Medicine, Yonsei University College of Medicine, Seoul 03722, Republic of Korea; jam764@yuhs.ac (J.K.);; 2 AIDS Research Institute, Yonsei University College of Medicine, Seoul 03722, Republic of Korea; 3 Department of Internal Medicine, Hanyang University Medical Center, Seoul 04763, Republic of Korea

**Keywords:** ChAdOx1 nCoV-19, adenovirus vaccines, adenoviridae, immunogenicity, vaccine

## Abstract

This study investigated the immunogenicity of, and reactogenicity to, the ChAdOx1 nCoV-19 vaccine according to pre-existing adenovirus immunity. Individuals scheduled for COVID-19 vaccination were prospectively enrolled in a tertiary hospital with 2400 beds from March 2020 onwards. Pre-existing adenovirus immunity data was obtained before ChAdOx1 nCoV-19 vaccination. A total of 68 adult patients administered two doses of the ChAdOx1 nCoV-19 vaccine were enrolled. Pre-existing adenovirus immunity was identified in 49 patients (72.1%), but not in the remaining 19 patients (27.9%). The geometric mean titer of S-specific IgG antibodies was statistically higher in individuals without pre-existing adenovirus immunity at several time points: before the second ChAdOx1 nCoV-19 dose (56.4 (36.6–125.0) vs. 51.0 (17.9–122.3), *p* = 0.024), 2–3 weeks after the second ChAdOx1 nCoV-19 dose (629.5 (451.5–926.5) vs. 555.0 (287.3–926.0), *p* = 0.049), and 3 months after the second ChAdOx1 nCoV-19 dose (274.5 (160.5–655.3) vs. 176.0 (94.3–255.3), *p* = 0.033). In the absence of pre-existing adenovirus immunity, systemic events were observed with higher frequency, especially chills (73.7% vs. 31.9%, *p* = 0.002). In conclusion, individuals without pre-existing adenovirus immunity showed a higher immune response to ChAdOx1 nCoV-19 vaccination and a higher frequency of reactogenicity to ChAdOx1 nCoV-19 vaccination was observed.

## 1. Introduction

Adenovirus (Ad)-derived vectors are currently the most commonly used vectors in gene therapy, cancer, and vaccine clinical trials worldwide [1]. Ad-derived vectors have several advantages, such as efficient transduction of many proliferating and quiescent cell types, packaging large amounts of foreign deoxyribonucleic acid (DNA), large scale production while meeting clinical good manufacturing practice standards, and rare integration into the host chromosome [2,3]. 

However, due to the high incidence of Ad infections in the general population, pre-existing Ad immunity may have an impact on therapeutic efficiency and safety [4,5]. Antibodies bound to the viral capsid mediate sequestration by Fc receptor-positive cells and may lead to vector clearance and poor tissue transduction [2,6,7]. Zak et al. report that responses of volunteers with pre-existing Ad5 neutralizing antibodies show drastically reduced inflammatory responses to the vaccine [8]. On the other hand, Varnavski et al. show that the presence of pre-existing anti-vector immunity is associated with increased mortality following systemic vector infusion [9].

Several approaches are being tested to overcome pre-existing Ad immunity, such as generation of chemically modified Ad5 capsids, generation of chimeric Ads, complete replacement of Ad5-based vaccine platforms with alternative (human and non-human origin) Ad serotypes, and Ad5 genome modification [10]. Nevertheless, in the case of the Janssen (Ad26) or ChAdOx1 nCoV-19 vaccine, the antibody response rate of the Ad vector vaccine is lower than that of the mRNA vaccine [11,12]. In the case of pre-existing Ad immunity, the immune response may be reduced when the Ad vector vaccine is administered compared with that of the control group.

Therefore, this study investigated the immunogenicity of and reactogenicity to the ChAdOx1 nCoV-19 vaccine according to pre-existing Ad immunity.

## 2. Materials and Methods

### 2.1. Study Design

**Figure 1:**
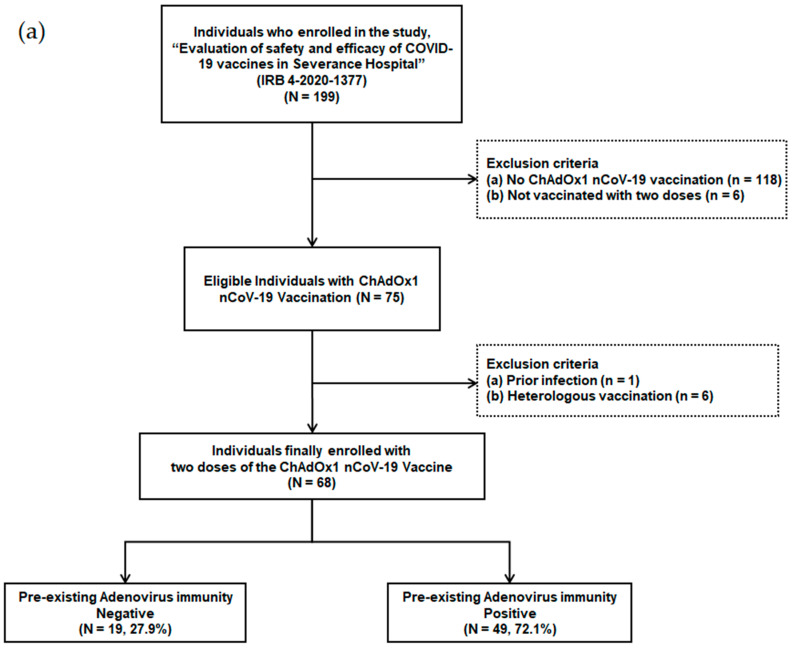

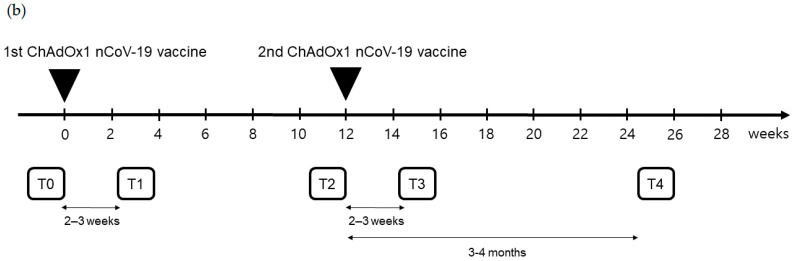
(**a**) Flow chart of individuals with ChAdOx1 nCoV-19 vaccination according to pre-existing adenovirus immunity; (**b**) Timetable of individuals with ChAdOx1 nCoV-19 vaccination.

Individuals scheduled for COVID-19 vaccination among patients and health care workers were prospectively enrolled in a tertiary hospital with 2400 beds starting from March 2020. The ChAdOx1 nCoV-19 vaccination was administered from 8 March 2021. Individuals who were not vaccinated with two doses (*n* = 6), individuals who received heterologous vaccination (*n* = 6), individuals who were not vaccinated with the ChAdOx1 nCoV-19 vaccine (*n* = 118), and individuals with a history of previous infection (*n* = 1) were excluded from the study (Figure 1a). A total of 68 adult individuals that received two doses of the ChAdOx1 nCoV-19 vaccine were finally enrolled.

The Institutional Review Board (IRB) of Yonsei University College of Medicine (IRB no. 4-2020-1377) approved this study. Informed consent was obtained from all participants. This study complied with the Declaration of Helsinki and Good Clinical Practice guidelines. 

### 2.2. Sample Selection

Peripheral blood samples were obtained at five time points: T0, before the first ChAdOx1 nCoV-19 dose; T1, 21.0 (18.0–24.0) days after the first ChAdOx1 nCoV-19 dose; T2, before the second ChAdOx1 nCoV-19 dose [71.0 (68.0–76.0) days after the first ChAdOx1 nCoV-19 dose]; T3, 16.0 (13.0–20.0) days after the second ChAdOx1 nCoV-19 dose; and T4, 103.0 (97.0–109.0) days after the second ChAdOx1 nCoV-19 dose (Figure 1b).

### 2.3. Adenovirus Titer Immunoassay 

Pre-existing Ad immunity data was obtained using the QuickTiter™ Adenovirus Titer enzyme-linked immunosorbent assay Kit (Cell BioLabs, San Diego, CA 92126, USA) at time point T0, before the first ChAdOx1 nCoV-19 dose [13]. HEK 293T cells (5 × 10^5^ cells/mL) were grown as monolayers in 96-well plates and incubated at 37 °C, 5% CO_2_ for 1 h. HEK 293T cells were infected with 10-fold serial dilution of T0 samples from enrolled patients or a 2-fold serial dilution of an Ad-β-gal positive control. After the immunoassay, the optical density at 450 nm was measured using a VersaMax Microplate Reader (Molecular Devices, LLC, San Jose, CA 95134, USA). Viral titers were calculated based on standard curves from Ad-β-gal positive control titrations. 

### 2.4. Immunogenicity of the ChAdOx1 nCoV-19 Vaccine

Immunogenicity data were obtained at each time point along the primary series of ChAdOx1 nCoV-19 vaccination. The humoral immune response was assessed by testing anti-SARS-CoV-2 spike (S) antibody titers in the serum using the Elecsys^®^ Anti-SARS-CoV-2 S assay Kit (Roche Diagnostics International Ltd., Rotkreuz, Switzerland). Plaque reduction neutralization tests (PRNT) were performed in duplicate using 24-well tissue culture plates (TPP Techno Plastic Products AG, Trasadingen, Switzerland) in a biosafety level 3 facility with Vero E6 TMRESS2 cells. Antibody titers were defined as the highest serum dilution that resulted in >50% (PRNT_50_) reduction in the number of virus plaques [14,15,16].

### 2.5. Reactogenicity to the ChAdOx1 nCoV-19 Vaccine

Solicited local and systemic adverse reactions were self-reported over 7 days after each dose, including rash, edema, vomiting, diarrhea, headache, fatigue, chills, muscle pain, joint pain, and fever. Unsolicited adverse events within 28 days after each dose and any adverse reactions leading to discontinuation were also reported. Reactogenicity severity was reported on a scale of 0–4: 0, none; 1, mild; 2, moderate; 3, severe; and 4, very severe. Serious adverse events were collected throughout the entire study period.

### 2.6. Statistical Analysis

Categorical variables were described by numbers and percentages, and continuous variables were expressed as median with interquartile range (IQR). Comparisons between groups were analyzed using the Chi-square and Fisher’s exact tests for categorical variables and the Mann–Whitney *U* test for continuous variables. *p* < 0.05 was considered significant. Statistical analysis was performed using GraphPad Prism 5 (GraphPad Software Inc.; San Diego, CA, USA) and IBM SPSS Statistics for Windows version 26 (IBM Corp., Armonk, NY, USA).

## 3. Results

### 3.1. Characteristics of Patients Who Received Two Doses of the ChAdOx1 nCoV-19 Vaccine

Out of 199 patients and health care workers, 68 individuals, who received two doses of the ChAdOx1 nCoV-19 vaccine, were finally enrolled (Figure 1a). These individuals were divided into two groups based on their pre-existing Ad immunity. Pre-existing Ad immunity was identified in 49 patients (72.1%), but not in the remaining 19 patients (27.9%).

The median age was 55 years (36–64), and 50.0% of patients were female (Table 1). The median body mass index was 22.1 (20.5–24.6) kg/m^2^. There were no significant differences in baseline comorbidities between the two groups. Out of the finally enrolled patients, 15 individuals (22.1%) had hypertension, 11 (16.2%) had diabetes mellitus, 2 (2.9%) had coronary artery occlusive disease, 2 (2.9%) had congestive heart failure, 6 (8.8%) had peripheral vascular disease, 3 (4.4%) had chronic kidney disease, 5 (7.4%) had chronic obstructive pulmonary disease, 4 (5.9%) had liver disease, and 1 (1.5%) had connective tissue disease. The median of Charlson comorbidity index was 3 (0–8). The time intervals of blood sampling based on ChAdOx1 nCoV-19 vaccination were not statistically different between the two groups (*p* = 0.951, *p* = 0.625, *p* = 0.143, and *p* = 0.134, respectively).

### 3.2. Immunogenicity of the ChAdOx1 nCoV-19 Vaccine

**Figure 2:**
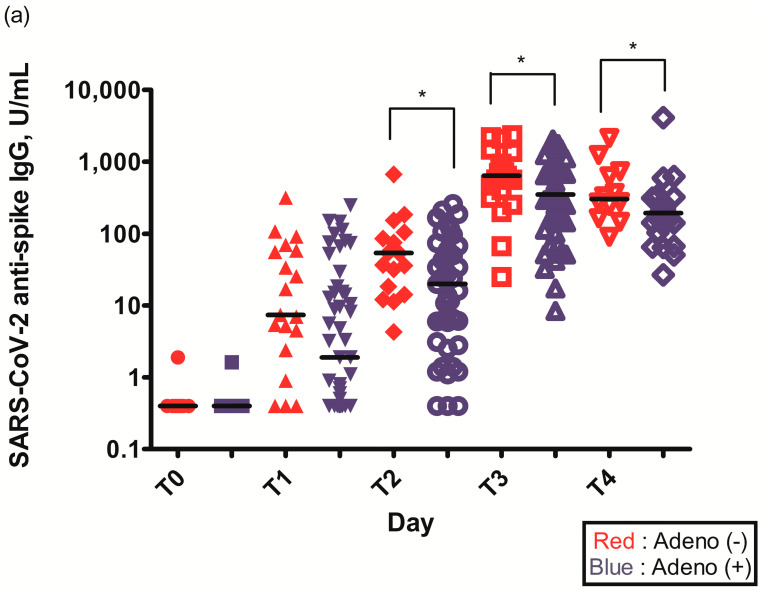

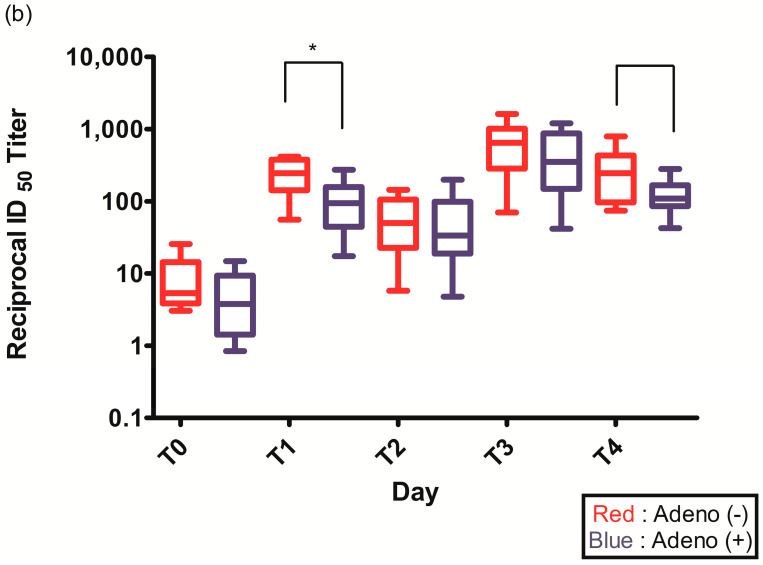
(**a**) Spike (S)-specific IgG titers after ChAdOx1 nCoV-19 vaccination according to pre-existing adenovirus immunity; (**b**) Plaque reduction neutralization test (PRNT_50_) data after ChAdOx1 nCoV-19 vaccination according to pre-existing adenovirus immunity; *, *p* < 0.05.

The geometric mean titer (GMT) of S-specific IgG antibodies 2–3 weeks after the first ChAdOx1 nCoV-19 dose (T1) was higher in individuals without pre-existing Ad immunity (29.6 (6.6–75.3) vs. 14.0 (1.6–84.5), *p* = 0.109) (Table 2). The GMT of S-specific IgG antibodies was statistically higher in individuals without pre-existing Ad immunity before the second ChAdOx1 nCoV-19 dose (T2) (56.4 (36.6–125.0) vs. 51.0 (17.9–122.3), *p* = 0.034), 2–3 weeks after the second ChAdOx1 nCoV-19 dose (T3) (629.5 (451.5–926.5) vs. 555.0 (287.3–926.0), *p* = 0.049), and 3 months after the second ChAdOx1 nCoV-19 dose (T4) (274.5 (160.5–655.3) vs. 176.0 (94.3–255.3), *p* = 0.033) (Figure 2a, Table 2).

The PRNT_50_ 2–3 weeks after the first ChAdOx1 nCoV-19 dose (T1) was statistically higher in individuals without pre-existing Ad immunity (245.2 (142.6–377.1) vs. 94.1 (44.4–157.5), *p* = 0.021) (Figure 2b, Table 2). The PRNT_50_ of other time points after ChAdOx1 nCoV-19 vaccination were higher in individuals without pre-existing Ad immunity but were not statistically different: before the second ChAdOx1 nCoV-19 dose (T2) (50.0 (22.7–105.4) vs. 33.6 (18.9–98.4), *p* = 0.774), 2–3 weeks after the second ChAdOx1 nCoV-19 dose (T3) (647.5 (283.2–1014.3) vs. 352.4 (149.2–867.9), *p* = 0.292), and 3 months after the second ChAdOx1 nCoV-19 dose (T4) (245.1 (96.5–432.2) vs. 108.8 (85.8–166.5), *p* = 0.065) (Figure 2b, Table 2).

The seroconversion rate was 75% after the first dose of ChAdOx1 nCoV-19 vaccine (84.2% vs. 71.4%, *p* = 0.359), but 100% after the second dose (Table 2).

### 3.3. Reactogenicity to the ChAdOx1 nCoV-19 Vaccine

**Figure 3:**
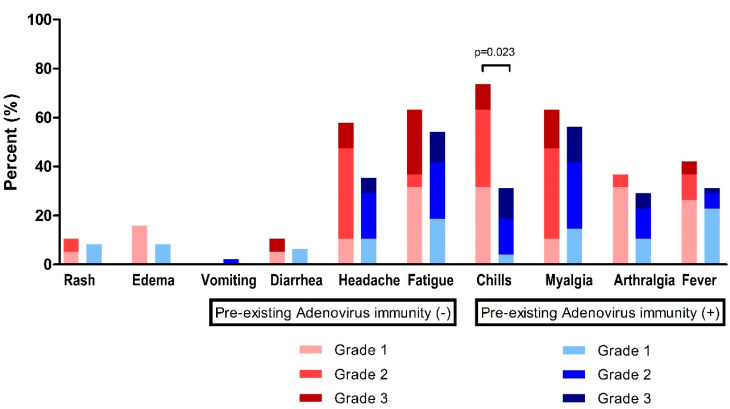
Reactogenicity to ChAdOx1 nCoV-19 vaccination according to pre-existing adenovirus immunity.

At time point T1 (2–3 weeks after the first ChAdOx1 nCoV-19 dose), fatigue (*n* = 38, 57.6%) and myalgia (*n* = 39, 59.1%) were the most frequently observed systemic events, followed by headache (*n* = 28, 42.4%), chills (*n* = 29, 43.9%), fever (*n* = 23, 34.8%), and arthralgia (*n* = 21, 31.8%) (Figure 3, Table 3). For these systemic adverse reactions, 48 patients (72.7%) took antipyretic medication (Table 3). 

In the absence of pre-existing Ad immunity, systemic events were observed with a higher frequency, but were not statistically different: fever, 42.1% vs. 31.9%, *p* = 0.431; headache, 57.9% vs. 36.2%, *p* = 0.106; and fatigue, 63.2% vs. 55.3%, *p* = 0.560 (Table 3). However, chills were significantly higher in individuals without pre-existing Ad immunity (73.7% vs. 31.9%, *p* = 0.002).

At time point T3 (2–3 weeks after the second ChAdOx1 nCoV-19 dose), there was no significant difference in the systemic reactions of both groups according to pre-existing Ad immunity (Supplemental Table S1).

## 4. Discussion

In this study, individuals without pre-existing Ad immunity had a higher immune response and increased reactogenicity to the ChAdOx1 nCoV-19 vaccine. In addition, the humoral response to the ChAdOx1 nCoV-19 vaccine lasted longer in individuals without pre-existing Ad immunity.

Adenovirus infection is a common cause of upper respiratory tract infections and can manifest in a range of clinical symptoms such as gastroenteritis, pneumonia, conjunctivitis, hepatitis, nephritis, and meningoencephalitis [17]. Adenoviruses have been identified in more than 100 genotypes and 50 serotypes, with the predominant serotypes varying by geographic regions and changing over time periods [18]. Most individuals have been exposed to one or more adenoviruses during their lifetime, and it typically leads to the development of long-lasting immunity against the specific serotypes that were encountered [19]. Among the adenoviruses one distinguishes to date more than 50 serotypes and >100 genotypes, the seroprevalence of neutralizing antibodies produced during the convalescent period after Ad infection varies depending on the subtype but is reported to be approximately 30–60% [20]. Kolavic-Gray SA et al. report that pre-existing serum neutralizing antibody has a protective effect for subsequent infection [20]. These findings are supported by clinical studies showing Ad seroprevalence increases with age and that more than 80% of Ad infections are observed in children under 4 years of age [21,22].

Adenoviral vectors have been employed in numerous applications of gene therapy, anti-cancer therapy, and vaccines due to their many advantages, which include efficient transduction of both dividing and non-dividing cells, the capacity to carry large DNA loads, large scale production capabilities meeting clinical good manufacturing practices, and low risk of integration into the host chromosome [2]. The influence of pre-existing immunity may vary depending on the specific application of adenoviral vectors. Although gene therapy and anti-cancer therapy employing adenoviral vectors aim to introduce therapeutic genes or drugs into cells, vaccines utilizing adenoviral vectors are intended to trigger an immune response against a particular pathogen or antigen [19]. Pre-existing immunity to Ad can take different forms, such as the presence of neutralizing antibodies, Ad-protein-specific T cell responses, and innate immune responses [3,10]. In particular, serum neutralizing antibodies can hinder the desired immune response against the targeted antigen. In some cases, pre-existing Ad immunity is associated with strong innate inflammatory responses within hours or increased mortality following systemic vector infusion [9,21]. However, in other cases, pre-existing Ad immunity shows significantly reduced inflammatory responses to the vaccine [8]. These immune responses affect not only the vector itself, but also transgene products and cascades [3]. Therefore, to overcome pre-existing Ad immunity, various strategies have been attempted.

Numerous methods are currently being explored to overcome pre-existing Ad immunity, including the production of chemically altered Ad5 capsids, the development of chimeric Ads, modification of Ad5 genomes, and substitution of Ad5-based vaccine platforms with alternative Ad serotypes [10]. In the case of the ChAdOx1 nCoV-19 vaccine, chimpanzee adenovirus is used as a vector to overcome pre-existing Ad immunity [23]. Chimpanzee adenovirus serotypes 63 (ChAd-63), ChAd-68, and ChAd-Y25, which are frequently used, exhibit approximately 90% sequence similarity with the human adenovirus species group E [24,25]. Additionally, up to 90% of residues in three-dimensional positions among ChAd-68, fowl adenovirus 1, Ad2, Ad4, and Ad5 were found to be closely matched when comparing their hexon models using crystallographic methods [26]. Therefore, the similarity of both the sequences and three-dimensional structures implies that the presence of pre-existing immunity to different subtypes via a common hexon structure may impact the immune response [24,26]. Xiang Z et al. reported that the immune response to the Ad5 vaccine was abolished in mice with pre-existing immunity to Ad5 and showed a reduced response in mice with pre-existing immunity to other human adenoviruses, such as serotypes 2, 4, 7, and 12 [27]. Furthermore, neutralizing antibodies against ChAd-68, ChAd-6, and ChAd-1 were frequently detected without any prior history of exposure, and their seroprevalence was reported to range from 21% to 44% [28,29,30]. The higher seroprevalence of neutralizing antibodies against chimpanzee adenoviruses might indicate the possibility of cross-species neutralization of these viruses [30,31]. These findings suggest that the host immune response may be affected by the cross-reaction of pre-existing Ad immunity despite the use of ChAdOx1. Specifically, this study found that the host immune response to the ChAdOx1 nCoV-19 vaccine was reduced when pre-existing immunity to adenoviruses was present.

In both groups, the seroconversion rate reached 100% after the second dose, but the overall median titer of S-specific IgG antibodies was lower in the group with pre-existing Ad immunity and was significantly reduced at time points T2–T4. For S-specific IgG titers at time point T2, seroconversion was not achieved in three individuals, all of whom had pre-existing Ad immunity. Seroconversion was achieved in all individuals 2–3 weeks after the second ChAdOx1 nCoV-19 dose (T3). However, at three months after second ChAdOx1 nCoV-19 dose (T4), both the median and minimum titers of S-specific IgG antibodies were reduced in the group with pre-existing Ad immunity. Furthermore, the ratio of S-specific IgG titers at time point T4 to S-specific IgG titers at time point T3 was lower in the group with pre-existing Ad immunity (31.7% (176.0/555.0) vs. 43.6% (274.5/629.5)). The fact that S-specific IgG titers in the group with pre-existing Ad immunity showed a lower peak and greater decline (68.3% vs. 56.4%) over the same period suggested that antibody waning might occur more quickly in these patients. This indicated that antibody response longevity was longer in the group without pre-existing Ad immunity.

In our study, the PRNT_50_ after ChAdOx1 nCoV-19 vaccination was also higher in individuals without pre-existing Ad immunity, and statistically significantly higher at 2–3 weeks after the first ChAdOx1 nCoV-19 dose (T1). The association between neutralizing antibody titer and clinical protection against COVID-19 is intricate and requires many considerations [32,33]. According to the findings of David S. Khoury and colleagues, neutralization titer serves as a significant predictor of both protective immune responses and the long-term dynamics of SARS-CoV-2 immunity [33]. The study also suggests that a decline in the neutralization titer post-immunization can result in a substantial loss of protection against SARS-CoV-2 infection, while protection against severe disease is expected to remain mostly intact [33].

Reactogenicity to the first ChAdOx1 dose (T1) was observed at a higher frequency in the group without pre-existing Ad immunity. In particular, systemic events, such as chills were significantly higher in individuals without pre-existing Ad immunity. However, reactogenicity to the second ChAdOx1 dose did not show a significant difference. The difference of immunogenicity after the first ChAdOx1 dose (T1) was also reduced after the second ChAdOx1 dose (T3 and T4). These observations suggested that repeated administration of adenoviral vectors reduces subsequent immune response. Successful gene delivery and expression were achieved upon repeated administration of adenoviral vectors, albeit at significantly reduced levels in comparison to the initial vector administration [34,35]. In the case of Ad5-vectored COVID-19 vaccine, pre-existing immunity to the Ad5 vector was associated with reduced humoral immune responses and lower occurrence of fever after vaccination [36].

Ad-derived vectors are used in various trials because of their advantages, such as efficient transduction, packaging of large amounts of foreign DNA, and rare integration into the host chromosome [2,3]. However, according to the results of recent population-based studies, the efficacy of Ad vector-based vaccines has decreased compared to mRNA COVID-19 vaccines, and mRNA COVID-19 vaccines are preferred as a booster vaccine for COVID-19 [11,12]. These results suggest that there are limitations to Ad vector-based vaccines due to the presence of pre-existing Ad immunity or repeated administration of Ad-derived vectors. Nevertheless, the fact that the seroconversion rate is increased by re-administration suggests that there is potential for improvement with a proper understanding of the immune response according to pre-existing Ad immunity.

This study has several limitations. First, because the sample size of this study was small, the statistical analysis was performed using a non-parametric test. To minimize the influence of confounding factors, the baseline characteristics and blood sampling times of the two groups were matched as closely as possible. Second, because the study was only conducted in Korea, various races could not be considered. However, since the study was conducted in a population with equal seroprevalence of Ad, the influence of confounding factors was also reduced. Third, this study only assessed the humoral immune response based on pre-existing Ad immunity. Further research examining the pre-existing and post-vaccination T cell response could offer additional insights into the subsequent immune response.

## 5. Conclusions

In conclusion, in individuals without pre-existing Ad immunity, the immune response to ChAdOx1 nCoV-19 vaccination was higher and reactogenicity to ChAdOx1 nCoV-19 vaccination was observed with a higher frequency. Seroconversion was achieved in all individuals after the second ChAdOx1 nCoV-19 dose; however, the humoral response to ChAdOx1 nCoV-19 vaccination lasted longer in individuals without pre-existing Ad immunity. Thus, for Ad-derived vaccination to have sufficient immune response and longevity, pre-existing Ad immunity must be evaluated.

## Figures and Tables

**Table 1:** Baseline characteristics of individuals with ChAdOx1 nCoV-19 vaccination according to pre-existing adenovirus immunity.

	Total (*n* = 68)	Adenovirus Immunity		*p* Value
		Negative (*n* = 19, 27.9%)	Positive (*n* = 49, 72.1%)	
Age (years)	55 (36–64)	40 (30–61)	56 (37–65)	0.158
Male sex (%)	34 (50.0%)	10 (52.6%)	24 (49.0%)	0.787
Body mass index (kg/m^2^)	22.1 (20.5–24.6)	23.0 (20.8–25.0)	21.5 (20.1–24.4)	0.403
Hypertension	15 (22.1%)	3 (15.8%)	12 (24.5%)	0.530
Diabetes mellitus	11 (16.2%)	1 (5.3%)	10 (20.4%)	0.163
Coronary artery occlusive disease	2 (2.9%)	2 (10.5%)	0 (0.0%)	0.075
Congestive heart failure	2 (2.9%)	1 (5.3%)	1 (2.0%)	0.484
Peripheral vascular disease	6 (8.8%)	3 (15.8%)	3 (6.1%)	0.338
Solid tumor	34 (50.0%)	8 (42.1%)	26 (53.1%)	0.590
Chronic kidney disease	3 (4.4%)	0 (0.0%)	3 (6.1%)	0.554
Chronic obstructive pulmonary disease	5 (7.4%)	2 (10.5%)	3 (6.1%)	0.614
Liver disease	4 (5.9%)	1 (5.3%)	3 (6.1%)	0.999
Connective tissue disease	1 (1.5%)	0 (0.0%)	1 (2.0%)	0.999
Peptic ulcer disease	2 (2.9%)	2 (10.5%)	0 (0.0%)	0.075
Charlson comorbidity index	3 (0–8)	0 (0–9)	4 (0–8)	0.880
Intervals (days)				
Interval between the first ChAdOx1 nCoV-19 dose and T1	21.0 (18.0–24.0)	21.5 (18.0–24.0)	20.0 (18.5–23.5)	0.951
Interval between the first ChAdOx1 nCoV-19 dose and T2	71.0 (68.0–76.0)	70.0 (67.5–74.5)	71.0 (68.0–76.5)	0.625
Interval between the second ChAdOx1 nCoV-19 dose and T3	16.0 (13.0–20.0)	15.5 (13.0–16.5)	18.0 (13.5–23.5)	0.143
Interval between the second ChAdOx1 nCoV-19 dose and T4	103.0 (97.0–109.0)	100.0 (96.0–105.0)	104.0 (97.5–118.5)	0.134

Continuous variables are described as median and interquartile range (IQR), and discrete variables are described as numbers (%). T0, before the first ChAdOx1 nCoV-19 vaccination; T1, 2–3 weeks after the first ChAdOx1 nCoV-19 dose; T2, before the second ChAdOx1 nCoV-19 dose; T3, 2–3 weeks after the second ChAdOx1 nCoV-19 dose; T4, 3 months after the second ChAdOx1 nCoV-19 dose.

**Table 2:** Humoral responses of individuals with ChAdOx1 nCoV-19 vaccination according to pre-existing adenovirus immunity.

Immunogenicity	Total (*n* = 68)	Adenovirus Immunity		*p* Value
		Negative (*n* = 19, 27.9%)	Positive (*n* = 49, 72.1%)	
PRNT_50_ ^a^ at T0	5.07 (3.04–9.55)	5.35 (3.84–14.39)	3.77 (1.43–9.35)	0.231
Spike (S)-specific IgG titers at T0	0.4 (0.4–0.4)	0.4 (0.4–0.4)	0.4 (0.4–0.4)	0.479
PRNT_50_ at T1	146.5 (57.3–271.5)	245.2 (142.6–377.1)	94.1 (44.4–157.5)	0.021 ^b^
S-specific IgG titers at T1	5.0 (0.4–31.6)	29.6 (6.6–75.3)	14.0 (1.6–84.5)	0.109
PRNT_50_ at T2	37.6 (19.3–100.1)	50.0 (22.7–105.4)	33.6 (18.9–98.4)	0.774
S-specific IgG titers at T2	31.9 (6.1–74.1)	56.4 (36.6–125.0)	51.0 (17.9–122.3)	0.034 ^c^
PRNT_50_ at T3	432.2 (163.5–904.8)	647.5 (283.2–1014.3)	352.4 (149.2–867.9)	0.292
S-specific IgG titers at T3	415.5 (165.3–831.0)	629.5 (451.5–926.5)	555.0 (287.3–926.0)	0.049 ^c^
PRNT_50_ at T4	117.8 (89.8–262.3)	245.1 (96.5–432.2)	108.8 (85.8–166.5)	0.065
S-specific IgG titers at T4	245.0 (141.3–339.0)	274.5 (160.5–655.3)	176.0 (94.3–255.3)	0.033 ^c^
Seroconversion rate				
After the first dose	51 (75.0%)	16 (84.2%)	35 (71.4%)	0.359
After the second dose	68 (100.0%)	19 (100.0%)	49 (100.0%)	

^a^ PRNT_50_: Plaque reduction neutralization test, highest serum dilution that resulted in >50% reduction in viral plaques. ^b^ The PRNT_50_ at T1 was statistically higher in individuals without pre-existing Ad immunity. ^c^ The geometric mean titer of S-specific IgG antibodies at T2, T3, and T4 was statistically higher in individuals without pre-existing Ad immunity. T0, before the first ChAdOx1 nCoV-19 dose; T1, 2–3 weeks after the first ChAdOx1 nCoV-19 dose; T2, before the second ChAdOx1 nCoV-19 dose; T3, 2–3 weeks after the second ChAdOx1 nCoV-19 dose; T4, 3 months after the second ChAdOx1 nCoV-19 dose.

**Table 3:** Reactogenicity to the first dose of ChAdOx1 nCoV-19 vaccine according to pre-existing adenovirus immunity.

Reactogenicity	Total (*n* = 68)	Adenovirus Immunity		*p* Value
		Negative (*n* = 19, 27.9%)	Positive (*n* = 49, 72.1%)	
Use of antipyretic medication	48 (72.7%)	15 (78.9%)	33 (70.2%)	0.471
Rash	6 (9.1%)	2 (10.5%)	4 (8.5%)	0.999
Edema	7 (10.6%)	3 (15.8%)	4 (8.5%)	0.401
Vomiting	1 (1.5%)	0 (0.0%)	1 (2.1%)	0.999
Diarrhea	5 (7.6%)	2 (10.5%)	3 (6.4%)	0.621
Headache	28 (42.4%)	11 (57.9%)	17 (36.2%)	0.106
Fatigue	38 (57.6%)	12 (63.2%)	26 (55.3%)	0.560
Chills	29 (43.9%)	14 (73.7%)	15 (31.9%)	0.002 ^a^
Myalgia	39 (59.1%)	12 (63.2%)	27 (57.4%)	0.669
Arthralgia	21 (31.8%)	7 (36.8%)	14 (29.8%)	0.577
Fever	23 (34.8%)	8 (42.1%)	15 (31.9%)	0.431

^a^ Chills were statistically higher in individuals without pre-existing Ad immunity. Solicited local and systemic adverse reactions are self-reported over 7 days after each dose. Reactogenicity severity is reported on a scale from 0–4: 0, none; 1, mild; 2, moderate; 3, severe; and 4, very severe.

## Data Availability

The data presented in this study are available on request from the corresponding author.

## Supplementary Materials

The following supporting information can be downloaded at: https://www.mdpi.com/article/10.3390/vaccines11040784/s1. Table S1. Reactogenicity to the second dose of ChAdOx1 nCoV-19 vaccine according to pre-existing adenovirus immunity.
