# Supplementary material for: Immunogenicity Differences of the ChAdOx1 nCoV-19 Vaccine According to Pre-Existing Adenovirus Immunity
Source: Vaccines (Basel). 2023 Apr 1;11(4):784. doi: 10.3390/vaccines11040784 (PMC10145356; doi:10.3390/vaccines11040784)
Supplement: Supplementary file 1 [file vaccines-11-00784-s001.zip › vaccines-2262052-supplementary.pdf]

Supplemental Table S1. Reactogenicity to the second dose of ChAdOx1 nCoV-19 vaccine according to pre-existing Adenovirus immunity.

	**Total (n = 67)**	**Adenovirus immunity**		***p* value**
		**Negative (n = 19, 28.4%)**	**Positive† (n = 48, 71.6%)**	
Use of antipyretic medication	33 (49.3%)	9 (47.4%)	24 (50.0%)	0.846
Rash	4 (6.0%)	1 (5.3%)	3 (6.3%)	0.999
Edema	3 (4.5%)	1 (5.3%)	2 (4.2%)	0.999
Headache	10 (14.9%)	5 (26.3%)	5 (10.4%)	0.132
Fatigue	24 (35.8%)	4 (21.1%)	20 (41.7%)	0.113
Chills	5 (7.5%)	1 (5.3%)	4 (8.3%)	0.999
Myalgia	10 (14.9%)	4 (21.1%)	6 (12.5%)	0.452
Arthralgia	1 (1.5%)	0 (0.0%)	1 (2.1%)	0.999
Fever	3 (4.5%)	1 (5.3%)	2 (4.2%)	0.999

*Solicited local and systemic adverse reactions are self-reported over 7 days after each dose.

*Reactogenicity severity is reported on a scale of 0–4: 0, none; 1, mild; 2, moderate; 3, severe; and 4, very severe.

†One patient with pre-existing Adenovirus immunity is not self-reported solicited local and systemic adverse reactions.
